# Automatic Pharyngeal Phase Recognition in Untrimmed Videofluoroscopic Swallowing Study Using Transfer Learning with Deep Convolutional Neural Networks

**DOI:** 10.3390/diagnostics11020300

**Published:** 2021-02-13

**Authors:** Ki-Sun Lee, Eunyoung Lee, Bareun Choi, Sung-Bom Pyun

**Affiliations:** 1 Medical Science Research Center, Ansan Hospital, Korea University College of Medicine, Ansan-si 15355, Korea; 2 Department of Physical Medicine and Rehabilitation, Anam Hospital, Korea University College of Medicine, Seoul 02841, Korea; euneun9204@naver.com (E.L.); brchoi614@gmail.com (B.C.); 3 Department of Biomedical Sciences, Korea University College of Medicine, Seoul 02841, Korea; 4 Brain Convergence Research Center, Korea University College of Medicine, Seoul 02841, Korea

**Keywords:** videofluoroscopic swallowing study, action recognition, deep learning, convolutional neural network, transfer learning

## Abstract

Background: Video fluoroscopic swallowing study (VFSS) is considered as the gold standard diagnostic tool for evaluating dysphagia. However, it is time consuming and labor intensive for the clinician to manually search the recorded long video image frame by frame to identify the instantaneous swallowing abnormality in VFSS images. Therefore, this study aims to present a deep leaning-based approach using transfer learning with a convolutional neural network (CNN) that automatically annotates pharyngeal phase frames in untrimmed VFSS videos such that frames need not be searched manually. Methods: To determine whether the image frame in the VFSS video is in the pharyngeal phase, a single-frame baseline architecture based the deep CNN framework is used and a transfer learning technique with fine-tuning is applied. Results: Compared with all experimental CNN models, that fine-tuned with two blocks of the VGG-16 (VGG16-FT5) model achieved the highest performance in terms of recognizing the frame of pharyngeal phase, that is, the accuracy of 93.20 (±1.25)%, sensitivity of 84.57 (±5.19)%, specificity of 94.36 (±1.21)%, AUC of 0.8947 (±0.0269) and Kappa of 0.7093 (±0.0488). Conclusions: Using appropriate and fine-tuning techniques and explainable deep learning techniques such as grad CAM, this study shows that the proposed single-frame-baseline-architecture-based deep CNN framework can yield high performances in the full automation of VFSS video analysis.

## 1. Introduction

Dysphagia is defined as a clinical symptom of difficulty swallowing foods [1]. Neurological, muscular, anatomical, and/or psychological factors may predispose a person to swallowing difficulty [2]. Swallowing for nutrition should include respiratory protective movements [3]. Hence, underlying health conditions may interact with dysphagia to produce aspiration, pneumonia, and/or respiratory compromise [4]. Moreover, dysphagia may interfere with nutrition, delay clinical recovery and even results in death if not diagnosed early and appropriately [5]. Therefore, earlier detection of dysphagia results in the earlier appropriate selection of a treatment method. This not only shortens the reestablishment of the overall health status but also reduces the overall rehabilitation efforts and costs [6,7].

Videofluoroscopy swallowing study (VFSS) or a modified barium swallow study, is considered the gold standard tool for studying the oral and pharyngeal processes for evaluating the swallowing process of dysphasia patients [8]. During the analysis of VFSS, patients are asked to swallow solid and liquid food mixed with radiopaque materials. Subsequently, through fluoroscopy, the video data of the swallowing motion is collected. Clinicians repeatedly analyze the recorded video to evaluate abnormalities associated with the swallowing process [9].

**Figure 1:**
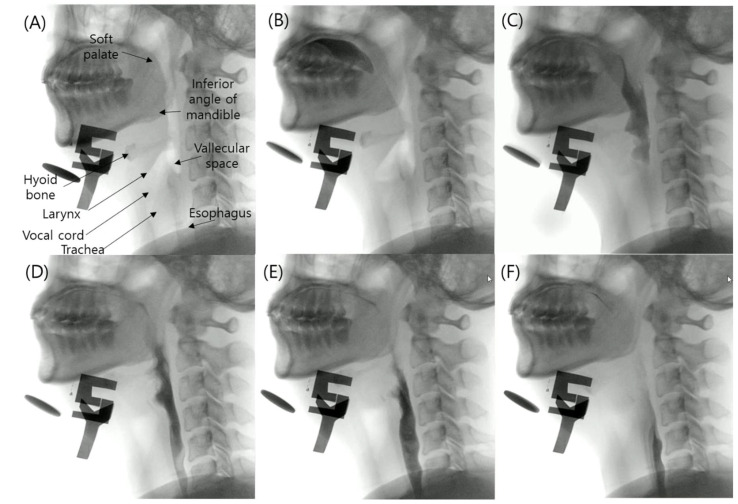
(**A**) Structural anatomy of normal swallowing of thick liquid bolus in (**B**) oral phase, (**C**–**E**) pharyngeal phase and (**F**) esophageal phase.

The swallowing process is generally categorized into three phases—the oral, pharyngeal and esophageal phases, as shown in Figure 1. During the oral phase, food is chewed and mixed with the saliva to form a bolus; next, the tongue pushes the bolus from the anterior to the posterior of the oral cavity. Subsequently, during the pharyngeal phase, the bolus is propelled from the oral cavity to the pharynx. At this moment, the hyoid bone and the larynx elevate and the epiglottis folds downward to protect the airway. This crucial point renders the pharyngeal phase a crucial phase of swallowing because it prevents the transport of the bolus to the airway system. After the airway is protected, the tail of the bolus exits through the opening of the upper esophageal sphincter. Finally, during the esophageal phase, the bolus passes down the esophagus to the stomach.

VFSS can illustrate the physiological process of the entire swallowing activity, including the motions of the jaws, tongue, palate, pharynx, larynx, esophagus and bolus of food [10]. Although VFSS is considered the standard for evaluating dysphasia and its video clips are collected as digital data, the evaluation of VFSS is a subjective interpretation based on visual inspection. A previous study reported that VFSS analysis is time consuming and laborious to a clinician [11]. Furthermore, another study reported that the consistency of the VFSS cannot be guaranteed owing to the subjectivity of the examiner when performing frame-by-frame analysis [12]. In particular, the recognition of the pharyngeal phase frames in VFSS by clinicians is crucial for shortening the examination time and revealing abnormalities in swallowing because aspiration or penetration occurs during the pharyngeal phase [13].

With recent efforts to obtain objective and consistent evaluations of VFSS image data, as well as with the rapid development of artificial intelligence (AI) research on medical imaging, several deep learning-based VFSS analysis methods have been suggested. In particular, inspired by the recent success of temporal action detection technology on action classification and action recognition in videos, such as three-dimensional convolutional networks (3DConvNets) [14,15], medical researchers have attempted to adopt these techniques to detect the pharyngeal phase in the VFSS [16]. However, 3DConvNets incur a significant computational cost and video clips of at least 16 frames with no large intervals as input data for training and prediction. Moreover, this method only manages the temporal window spanning for 512 frames at the least (approximately 17 s) [17]. Because the pharyngeal phase in the entire long VFSS videos occurs during the short frame sequence, a recent study reported that this cutting-edge deep learning technology may present limitations in recognizing activity during short frames in long-sequence videos [17,18].

Therefore, to suggest a simple but practical computer-aided detection system using generic deep learning technology, this study proposes a single-frame-baseline-architecture-based [19] convolutional neural network (CNN) framework that recognizes the occurrence of pharyngeal phase in every frame in VFSS videos.

## 2. Materials and Methods

### 2.1. Experimental Design

**Figure 2:**
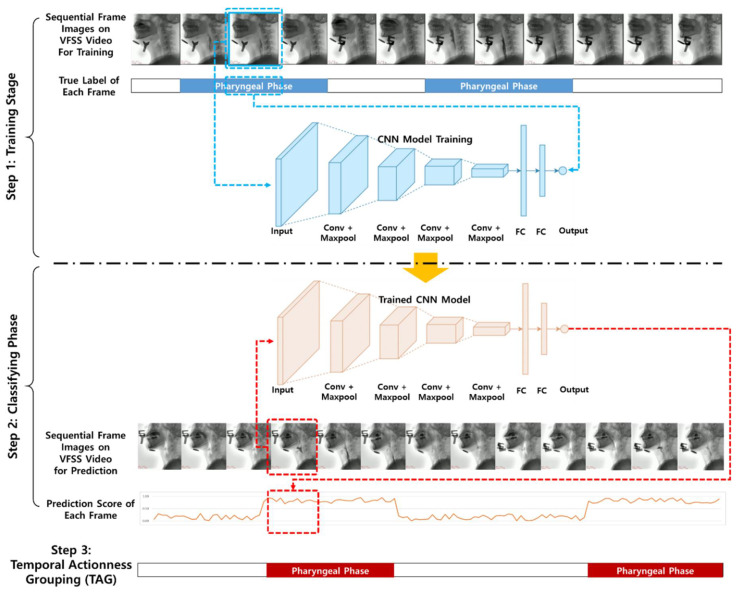
Conceptual diagram of framework proposed herein.

Figure 2 shows a conceptual diagram of the framework proposed herein.

To recognize the pharyngeal phase in a long frame of raw VFSS videos, this study proposes a framework composed of three stages: training, classifying and temporal action grouping stages. In the first training stage, a CNN model is trained by a dataset, where each frame is labeled whether it is the pharyngeal phase. In the second classification stage, each frame in the test dataset video is classified using a predicted score (0.0–1.0) regardless of whether each image corresponds to the pharyngeal phase using the trained CNN model. In the third stage, we integrate the classification results on each frame using the sliding window technique to recognize the pharyngeal phase in untrimmed VFSS videos.

### 2.2. Datasets

The VFSS video data were taken from all 54 subjects who visited the Department of Rehabilitation Medicine at Korea University Anam Hospital from 1 March to 30 June, 2020, who were experiencing subjective swallowing difficulties. The subjects were 19 to 94 years old (mean age 70.67 ± 14.73 years) and included 29 men and 25 women.

The collected VFSS dataset was recorded by rehabilitation medicine specialists who performed the VFSS based on the standard protocol [9]. During the VFSS, each subject was seated upright laterally in front of a fluoroscope and swallowed each of the following six substances that were mixed with diluted radio-opaque barium: 2 and 5 mL of liquid (orange juice), thick liquid (yogurt), semi-solid (boiled rice) and solid (rice). The radiological images of the lateral head and neck areas were sequentially recorded as a digital video file during the entire VFSS. The frame rate of the videos that had been collected was 30 frames per second. Because each subject swallowed six types of substances, 324 video clips were collected, including one pharyngeal phase. The length of video clips varied from 156 frames (5.2 s) to 2031 frames (67.7 s) with average 614.5 frames (20.5 s).

The entire collected video clips were randomly segmented into training and testing sets at a ratio of 80:20. In order to avoid over-estimation, the division was performed on a subject basis. Consequently, among 54 subjects (324 clips; 234,906 frames), 43 subjects (258 clips; 187,440 frames) were used for training and 11 subjects (66 clips; 47,466) were used for testing.

This study was conformity with the Declaration of Helsinki and Ethical Guidelines for Medical and Health Research Involving Human Subjects (https://www.wma.net/policies-post/wma-declaration-of-helsinki-ethical-principles-for-medical-research-involving-human-subjects/, accessed on 14 September 2020). Because this study was designed as retrospective study, the requirement to obtain informed consent was waived. This study was approved by the Institutional Review Board of the Korea University Medical Center (IRB No. 2021AN0019) and carried out according to the guidelines of the committee.

### 2.3. CNN

In deep learning, the CNN (or ConvNet) is a class of deep neural network that is the most typically applied in analyzing visual images [20]. CNNs can extract the relevant features from images for classification tasks. CNNs are composed of convolutional layers that are groups of filters. One visualization is to obtain an input image that maximizes the activation of a particular filter. This provides insight into the learning of a particular filter within the CNN. This method can be extended to the final dense layer to visualize the features that are important for a particular output class.

**Figure 3:**
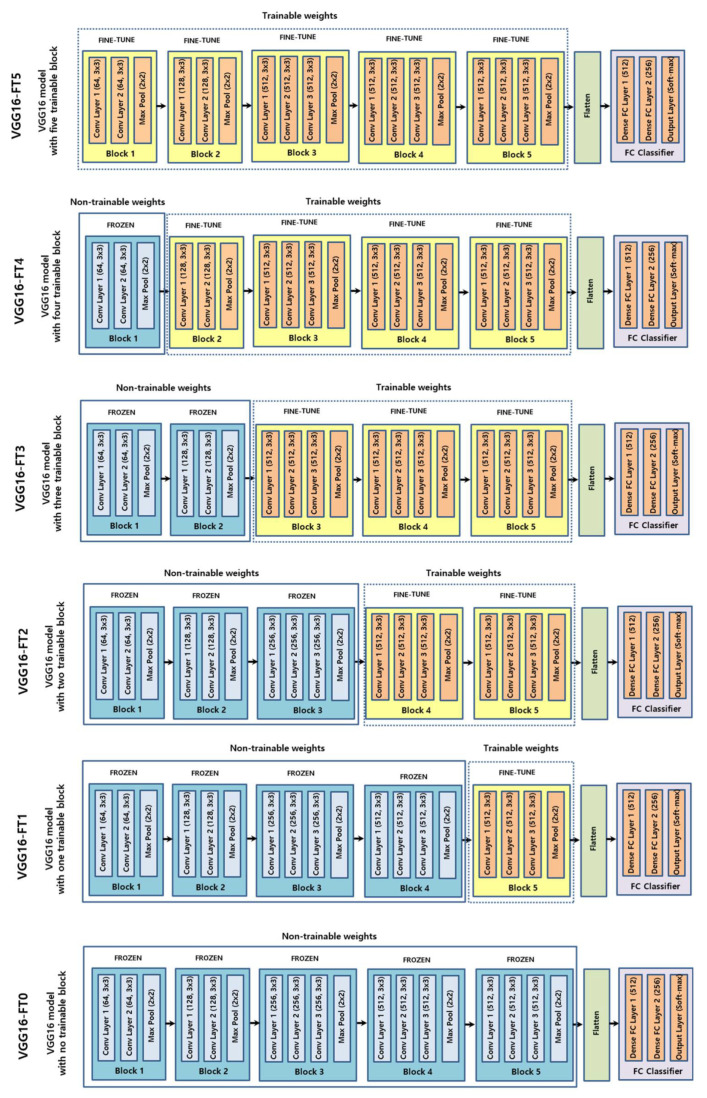
Schematic diagram of six experimental groups based on fine-tuning degree in VGG-16 backbone convolutional neural network (CNN).

This experiment was conducted using six different CNNs with different degrees of fine-tuning using VGG-16 [21] as the base CNN. VGG-16 is a pre-trained CNN developed from the Visual Geometry Group, Department of Engineering Science, University of Oxford. The VGG architecture has been widely applied and considered as a state-of-the-art architecture in both general and medical fields for various vision tasks, such as image feature extraction, image classification or object detection [22]. In VGG-16, 224 × 224 images are passed through five blocks of convolutional layers, where each block is composed of increasing numbers of 3 × 3 filters. In the five blocks, the first two blocks comprise two Conv layers, each followed by ReLU and MaxPool layers and the last three layers comprise three Conv layers, each followed by ReLU and MaxPool layers. The five blocks of convolutional layers are followed by two fully connected layers. The final layer is a soft-max layer that outputs class probabilities. Figure 3 shows the six experimental deep CNN groups, the schematic diagrams of the layer composition and the fine-tuning degree of VGG-16.

When the training dataset is relatively small, transferring a CNN pretrained by a large annotated dataset and fine-tuning it for a specific task can be an efficient method for achieving acceptable goals and lower training costs [23]. Although the classification of each frame image from VFSS videos differs from object classification and natural images, they can share similar learned features [24]. During transfer learning with a deep CNN via fine-tuning, weights in the CNN models were initialized based on pretraining on a general image dataset. However, some of the last blocks or layers in the CNN were unfrozen and learnable; therefore, their weights were updated in each training step. In this study, the VGG-16 used in this study as a backbone neural network comprised five blocks. Therefore, fine-tuning was performed in six approaches that were unfrozen sequentially from 0 to 5 blocks starting from the last block, depending on the number of unfrozen blocks. Consequently, VGG-16 was segmented into six subgroups according to the fine-tuning degree.

### 2.4. Training

**Figure 4:**
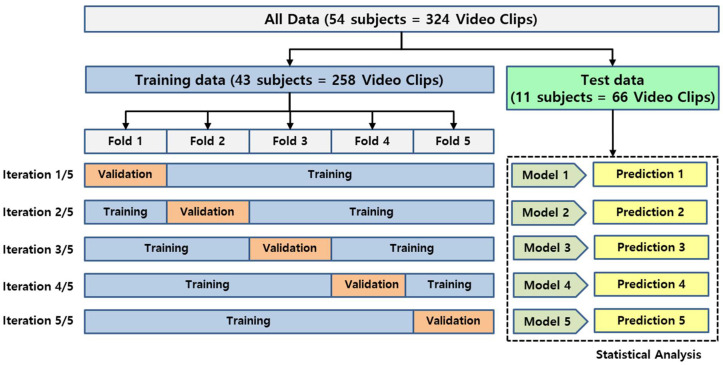
Overview of five-fold cross validation applied in this study.

The 258 video clips selected as the training dataset were randomly segmented into five folds to perform five-fold cross validation to evaluate the model training while avoiding overfitting or bias [25]. During each iteration, the dataset was independently partitioned into training and validation sets with a 80:20 ratio. The selected fold as validation set was a completely independent from the other folds as training and was used to evaluate the training performance during the training. After one iteration was completed, the other independent fold was used as a validation and the previous validation fold was reused as part of the training fold to evaluate the training performance. An overview of the five-fold cross validation conducted in this study is presented in Figure 4.

The training process above was repeated for all 12 experimental groups (Figure 3). All deep CNN models were trained, validated and evaluated on an NVIDIA DGX Station^TM^ (NVIDIA Corporation Santa Clara, CA, USA) with an Ubuntu 18 operating system, 256 GB of system memory and four NVIDIA Telsa V100 GPU. All the experiments were performed using the Keras [26] library and TensorFlow [27] backend engine. The initial training rate of each model was 0.00001. A ReduceLROn-Plateau method was employed because it reduces the learning rate when it stops improving the training performance. The RMSprop algorithm was used as the solver. After training all the five-fold deep CNN models, the best model was identified by testing using the test dataset.

### 2.5. Performance Evaluation

Three specialists in rehabilitation medicine annotated and validated the pharyngeal phase occurrence. They annotated the start and end frames of all occurrences of the pharyngeal phase in all experimental VFSS video clips. According to medical criteria [28,29], the beginning of the pharyngeal phase is defined as the moment when the head of the bolus is propelled to the pharynx, when the soft palate elevates and presses against the posterior wall of the pharynx. The end of the pharyngeal phase is defined as the point when the tail of the bolus exits through the opening of the upper esophageal sphincter.

To comprehensively evaluate the recognition performance of the pharyngeal phase on the test dataset, the accuracy, sensitivity, specificity, false positive rate (FPR), false negative rate (FNR), positive prediction value (PPV), Negative Prediction Value (NPV), diagnostic odds ratio (DOR), area under the receiver operating characteristic curve (AUC), Matthews correlation coefficient (MCC) and kappa were calculated as follows:

$$\mathrm{Accuracy}\;\left(\mathrm{ACC}\right)=\;\frac{TP+TN}{TP+TN+FN+FP}$$

$$\mathrm{Sensitivity}\;\left(\mathrm{True}\;\mathrm{Positive}\;\mathrm{Rate},\;\mathrm{TPR}\right)=\;\frac{TP}{TP+FN}$$

$$\mathrm{Specificity}\;\left(\mathrm{True}\;\mathrm{Negative}\;\mathrm{Rate},\;\mathrm{TNR}\right)=\;\frac{TN}{TN+FP}\;\mathrm{False}\;\mathrm{Positive}\;\mathrm{Rate}\;\left(\mathrm{FPR}\right)=\;\frac{FP}{FN+TN}\;\mathrm{False}\;\mathrm{Negative}\;\mathrm{Rate}\;\left(\mathrm{FNR}\right)=\;\frac{FN}{FN+TP}$$

$$\mathrm{Positive}\;\mathrm{Prediction}\;\mathrm{Value}\;\left(\mathrm{PPV}\right)=\;\frac{TP}{TP+FP}\;\mathrm{Negative}\;\mathrm{Prediction}\;\mathrm{Value}\;\left(\mathrm{NPV}\right)=\;\frac{TN}{TN+FN}\;\mathrm{Diagnostic}\;\mathrm{Odds}\;\mathrm{Ratio}\;\left(\mathrm{DOR}\right)=\;\frac{\left(TP/FN\right)\;}{\left(FP/TN\right)}\;\mathrm{Matthew}’s\;\mathrm{correlation}\;\mathrm{coefficient}\left(\mathrm{MCC}\right)=\;\frac{TP\times TN-FP\times FN\;}{\sqrt{\left(TP+FP\right)\left(TP+FN\right)\left(TN+FP\right)\left(TN+FN\right)}}$$

$$kappa=\;\frac{p_{0}-p_{e}}{1-p_{e}}$$

$$p_{0}=\;\frac{TP+TN}{TP+TN+FP+FN}\;p_{e}=\;\frac{\left(TP+FN\right)\times \left(TP+FP\right)+\left(FP+TN\right)\times \left(FN+TN\right)}{{\left(TP+TN+FP+FN\right)}^{2}}.$$

TP and FP denote the number of correctly and incorrectly predicted frame images from the entire video clip in the test dataset, respectively. Similarly, TN and FN represent the number of correctly and incorrectly predicted frame images from the entire video clip in the test dataset, respectively.

**Figure 5:**
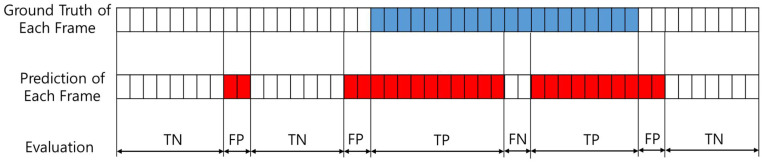
Conceptual diagram of pharyngeal phase recognitions results in test video fluoroscopic swallowing study (VFSS) video clip. Blue boxes mean pharyngeal phase frames labelled by specialists in VFSS video clips. Red boxes mean pharyngeal phase frames predicted by deep CNN model in VFSS video clips. TN: true negative; FP: false positive; TP: true positive; FN: false negative.

A sample of the evaluation process is shown in Figure 5. In one of the video clips in the test dataset, true pharyngeal phase-labeled frames existed in the video clip; predicted pharyngeal phase-labeled frames with confidence scores by the trained CNN model existed as well. In the evaluation process, any true labeled frame (true pharyngeal phase frame) that is not predicted as the pharyngeal phase frame by the trained CNN is counted as an FN and any false labeled frame (not pharyngeal phase frame) that is predicted as a pharyngeal phase frame by the trained CNN is counted as an FP.

## 3. Results

**Figure 6:**
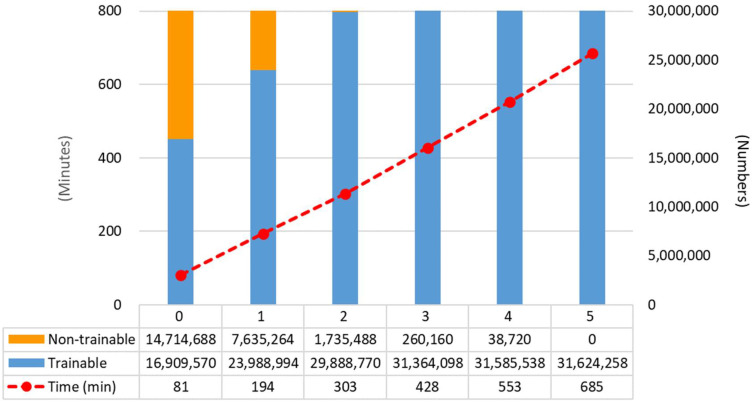
The number of trainable parameters (Trainable), the number of non-trainable parameters (Non-trainable) and the total training time (Time) of each experimental group in this study.

This study was conducted using experimental groups according to the degree of fine-tuning of one backbone deep CNN (VGG16). For each experimental group according to the number of trainable blocks (0 to 5) of the backbone deep CNN (VGG-16) used in this study, the number of non-trainable parameters, number of trainable parameters and training time of each experimental group are shown in Figure 6.

### 3.1. Classification Performance

**Figure 7:**
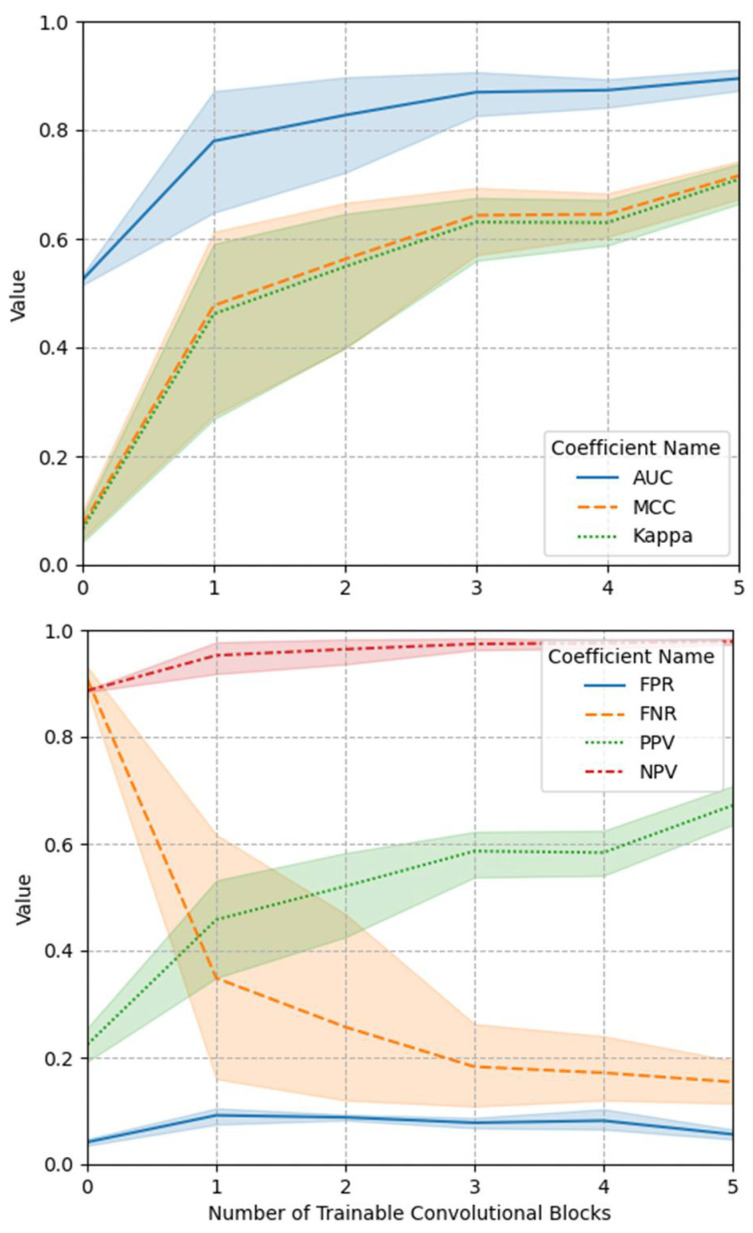
The changes of some indexes for model reliability (area under the receiver operating characteristic curve (AUC), Matthews correlation coefficient (MCC) and Kappa) and prediction scores (false positive (FPR), false negative (FNR), positive prediction (PPV) and negative prediction (NPV)) according to the numbers of trainable blocks in the deep CNN (VGG-16).

**Figure 8:**
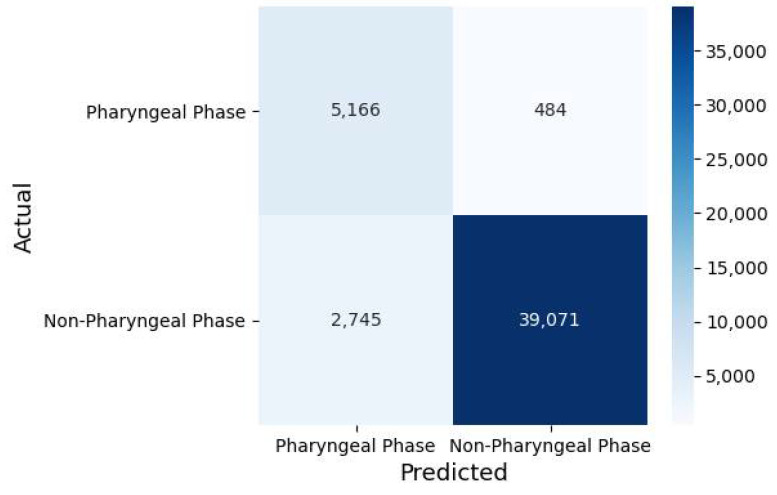
Confusion matrix of best performing classification model (fifth iteration of VGG16-FT5) in this study.

Table 1, Figure 7 and Figure 8 demonstrate the summarized prediction performance of each experimental group for recognizing the pharyngeal phase frames in the test VFSS video clips. In particular, Figure 7 depicts the changes of some indexes for model performance, reliability and prediction score according to the numbers of trainable blocks in the deep CNN (VGG-16).

Among all experimental groups, fine-tuned with all blocks of the VGG-16 model (VGG16-FT5) achieved the highest recognizing performance, that is, the accuracy of 93.20 (±1.25)%, sensitivity of 84.57 (±5.19)%, specificity of 94.36 (±1.21)%, FPR of 5.64 (±5.64)%, FNR of 15.43 (±5.19)%, PPV of 67.19 (±4.98)%, NPV of 97.84 (±0.72)%, DOR of 104.9054 (±36.92), AUC of 0.8947 (±0.0269), MCC of 0.716 1 (±0.0482) and Kappa of 0.7093 (±0.0488). All performance metrics values generated through 5-fold cross validation of each experimental group are presented in Supplementary Table S1.

### 3.2. Interpretation of Model Decision Using Grad-CAM

**Figure 9:**
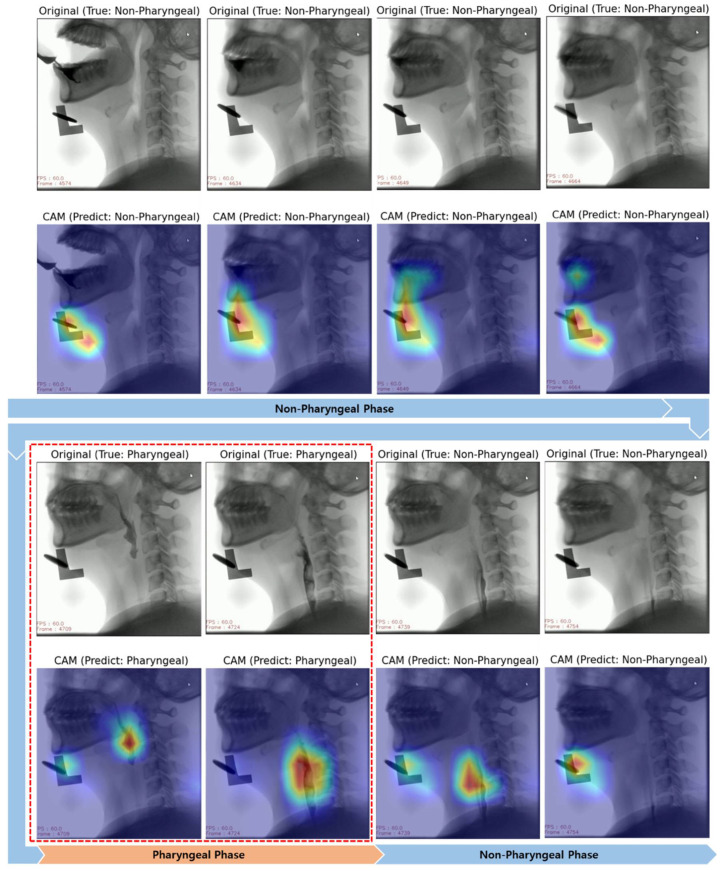
Sample frame images of original and gradient-weighted class activation mapping (Grad-CAM) correctly predicted by the best performing classification model (VGG16-FT5) in this study.

**Figure 10:**
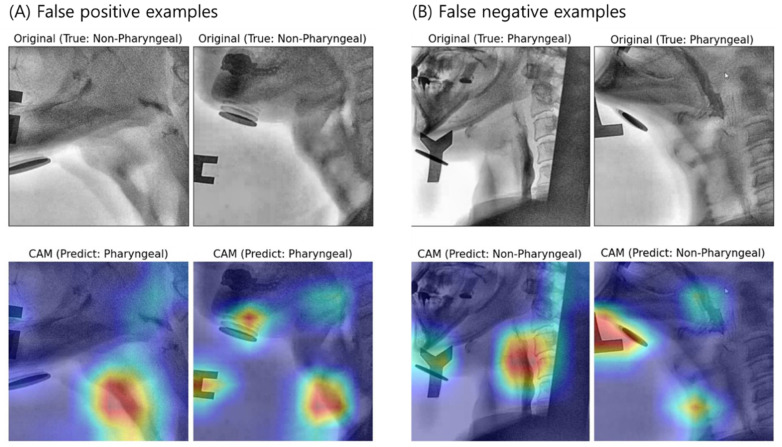
Sample images of original and Grad-CAM presumed to be falsely positively predicted (**A**) and falsely negatively predicted (**B**) by the best performing classification model (VGG16-FT5).

Figure 9 and Figure 10 show examples of visualized interpretation of predictions using deep CNN models in this study. In each example, the color heat map present areas that were most affected by the classification of the deep CNN model. Figure 9 shows a representative example of correctly classified cases for the pharyngeal phase in a VFSS video clip using the VGG16-TF5 CNN model that yielded the best classification performance. Figure 10 shows representative examples of FN and FP classifications, respectively.

## 4. Discussion

An automatic recognition of the pharyngeal phase frame from VFSS videos may be useful for the clinical analysis of VFSS. Clinically, the analysis of the pharyngeal phase in the VFSS video is important to the entire swallowing process, as it can identify any abnormalities that can result in serious medical complications, such as aspiration pneumonia or asphyxia [30]. To assess the pharyngeal phase in VFSS images, clinicians manually search for the pharyngeal phase in VFSS images through visual inspection. A deep-learning-based simple novel framework is proposed herein to automatically recognize pharyngeal phase frames in untrimmed VFSS video clips. A few previous studies with similar experimental purposes demonstrated high performances by using machine learning techniques [18] or 3D CNNs [16]. However, in the case of the use of 3D CNN, it is difficult to prepare a data set that combines temporal data with 2D images for the ground truth data for training and also it has high training costs due to complex algorithms. Although a single-frame-baseline architecture was used in this study, that is, the most basic architecture among large-scale video classification techniques using the CNN [19], the proposed framework showed the possibility to achieve a substantial level of discriminant ability (AUC = 0.8947 (±0.0269)) for identifying pharyngeal phase frames in VFSS video without unnecessary manual work.

### 4.1. Fine-Tuning Degree of Deep CNN

The CNN model learned from pre-training a large natural image dataset that can be used to classify common images but cannot be well utilized for specific classification tasks of medical images. However, based on a previous study that described the effects and mechanisms of fine-tuning on deep CNNs, when setting certain convolutional layers of a deep CNN model be trainable, the CNN model can be further specialized for specific classifying tasks [24,31]. Especially, the earlier layers of a pretrained CNN contain generic features that could be useful for many classification tasks; however, later layers of a pretrained CNN progressively contain more specialized features to the details of the classes contained in the original dataset. Using these characteristics, when the parameters of the early layers are preserved and that in later layers are updated during the training of new datasets, the CNN model can be effectively used in new classification tasks. In conclusion, setting the parameters in later layers of pre-trained CNN is trainable through the new dataset can improve the prediction performance and accuracy in the new classification task. This is known as the fin-tuning technique. Although the target medical image and the analysis purpose are different, the results are similar to those of previous studies [32,33,34] using the transfer learning of a deep CNN via fine-tuning.

As shown in Figure 7, as the trainable parameter increased, model performance (AUC) and model reliability (MCC and Kappa) increased. In particular, it was shown that as the trainable parameter increased, the PPV increased and the FNR decreased, thereby increasing the classification performance of the model. In particular, as the trainable parameter increases, the negative prediction (NPV) or false positive (FPR) hardly changes, whereas the positive prediction (PPV) increases and the false negative (FNR) decreases, thereby increasing the classification performance of the model. Can. This is expected to be due to data imbalance as the number of pharyngeal phase frames among the total number of VFSS video frames is relatively smaller than that of non-pharyngeal phase frames.

### 4.2. Visual Interpretation Using Grad-CAM

Grad-CAM uses the gradient information flowing into the last convolutional layer of the deep CNN to understand the significance of each neuron for making decisions [35]. For a qualitative evaluation of classification, the Grad-CAM technique was used in this study. In the CNN model, which demonstrated the best classification performance (Figure 9), image feature points for each class were specified for each frame in a VFSS video clip. In particular, it was confirmed that the food mass was automatically recognized as the pharyngeal phase when it was in the pharyngeal cavity. This shows that the CNN automatically classifies the pharyngeal phase and the non-pharyngeal phase without prior work, such as object labeling of food bolus in each frame image of VFSS video clips. However, in other swallowing phases (non-swallowing moment, oral phase and esophageal phase), it was classified as a non-pharyngeal phase through the recognition of non-biological markers rather than other biological markers. It is assumed that for images of unlabeled subsets other than the pharyngeal phase, the deep CNN algorithm generated hidden stratification [36].

When the pharyngeal phase is recognized as a non-pharyngeal phase (False Positive), the pharyngeal cavity was not imaged in the x-ray area as the patient moves and thus it was determined to be a similar part to the pharyngeal cavity (Figure 10A). Even in the pharyngeal phase, when the non-pharyngeal phase (False Negative) was recognized as a large foreign body in the x-ray area, there was a large patient motion, such as a large foreign body or the jaw being lifted too high unlike other patients (Figure 10B).

### 4.3. Limitations

This study has a limitation in that the sample size estimation method was not applied as it has only a limited set of data due to the characteristics of medical images that are limited to large-scale data collection. In addition, this study has a limitation in that it does not perform random sampling related to dataset sampling and uses sample of convenience that only uses VFSS videos taken at a certain time. The limitation of such a sampling method was mentioned in the previous literature [37], such as an inability to generalize the results of the survey to the population as a whole. Therefore, there is the possibility of under-or over-representation of the population in this study.

## 5. Conclusions

A single-frame-baseline-architecture-based simple CNN for recognizing pharyngeal phase in untrimmed VFSS video clips is presented here and the following conclusions are drawn. When using deep CNNs for recognizing the pharyngeal phase in VFSS video clips, by applying transfer learning technique to a deep CNN for classification, an appropriate fine-tuning degree was required. In addition, in the case of image classification using a deep CNN, classification must be evaluated qualitatively using visual interpretation methods such as the Grad-CAM technique to identify whether an appropriate classification has occurred based on the correct reason. The single-frame-baseline-architecture-based simple CNN using the factors above demonstrated the possibility of yielding high performances in the full automation of VFSS video analysis. However, this study was conducted based on images taken only in one institution and may have limitations compared to studies using multi-center and multiple imaging devices.

## Figures and Tables

**Table 1:** Performance metrics of experimental groups of this study.

Number of Fine-Tuning Blocks	0	1	2	3	4	5
Accuracy (ACC)	0.8551 (±0.0046)	0.8776 (±0.0214)	0.8918 (±0.0298)	0.9095 (±0.0184)	0.9075 (±0.0176)	0.9320 (±0.0125)
Sensitivity (TPR)	0.0914 (±0.0313)	0.6514 (±0.3040)	0.7429 (±0.2312)	0.8171 (±0.1061)	0.8286 (±0.0833)	0.8457 (±0.0519)
Specificity (TNR)	0.9583 (±0.0079)	0.9081 (±0.0207)	0.9120 (±0.0069)	0.9220 (±0.0129)	0.9181 (±0.0242)	0.9436 (±0.0121)
FPR	0.0417 (±0.0079)	0.0919 (±0.0207)	0.0880 (±0.0069)	0.0780 (±0.0129)	0.0819 (±0.0242)	0.0564 (±0.0121)
FNR	0.9086 (±0.0313)	0.3486 (±0.3040)	0.2571 (±0.2312)	0.1829 (±0.1061)	0.1714 (±0.0833)	0.1543 (±0.0519)
PPV	0.2240 (±0.0437)	0.4580 (±0.1224)	0.5204 (±0.1064)	0.5863 (±0.0576)	0.5835 (±0.0561)	0.6719 (±0.0498)
NPV	0.8864 (±0.0028)	0.9526 (±0.0378)	0.9641 (±0.0308)	0.9740 (±0.0149)	0.9756 (±0.0111)	0.9784 (±0.0072)
DOR	2.29 (±0.64)	32.40 (±23.87)	52.27 (±34.02)	74.00 (±44.04)	63.63 (±20.95)	104.91 (±36.92)
AUC	0.5249 (±0.0127)	0.7798 (±0.1428)	0.8274 (±0.1166)	0.8696 (±0.0546)	0.8734 (±0.0361)	0.8947 (±0.0269)
MCC	0.0739 (±0.0343)	0.4771 (±0.2185)	0.5626 (±0.1803)	0.6432 (±0.0836)	0.6452 (±0.0500)	0.7161 (±0.0482)
Kappa	0.0661 (±0.0323)	0.4616 (±0.2107)	0.5487 (±0.1720)	0.6305 (±0.0796)	0.6297 (±0.0528)	0.7093 (±0.0488)

## Supplementary Materials

The following are available online at https://www.mdpi.com/2075-4418/11/2/300/s1, Table S1: Performance metrics of experimental groups of this study.
