# Supplementary material for: Automatic Pharyngeal Phase Recognition in Untrimmed Videofluoroscopic Swallowing Study Using Transfer Learning with Deep Convolutional Neural Networks
Source: Diagnostics (Basel). 2021 Feb 13;11(2):300. doi: 10.3390/diagnostics11020300 (PMC7918932; doi:10.3390/diagnostics11020300)
Supplement: Supplementary file 1 [file diagnostics-11-00300-s001.pdf]

Supplementary Materials

Table S1. Performance metrics of experimental groups of this study.

Number of Fine-Tuning Blocks	Iteration Number of Cross Validation	ACC	SEN	SPE	FPR	FNR	PPV	NPV	DOR	AUC	MCC	Kappa
0	1	0.8571	0.0571	0.9653	0.0347	0.9429	0.1818	0.8834	1.68	0.5112	0.0382	0.0318
	2	0.8571	0.0571	0.9653	0.0347	0.9429	0.1818	0.8834	1.68	0.5112	0.0382	0.0318
	3	0.8605	0.1143	0.9614	0.0386	0.8857	0.2857	0.8893	3.21	0.5378	0.1151	0.1022
	4	0.8503	0.1143	0.9498	0.0502	0.8857	0.2353	0.8881	2.44	0.5320	0.0889	0.0824
	5	0.8503	0.1143	0.9498	0.0502	0.8857	0.2353	0.8881	2.44	0.5320	0.0889	0.0824
1	1	0.8469	0.1429	0.9421	0.0579	0.8571	0.2500	0.8905	2.71	0.5425	0.1092	0.1043
	2	0.8639	0.6000	0.8996	0.1004	0.4000	0.4468	0.9433	13.44	0.7498	0.4415	0.4351
	3	0.8912	0.8286	0.8996	0.1004	0.1714	0.5273	0.9749	43.31	0.8641	0.6047	0.5839
	4	0.8980	0.8000	0.9112	0.0888	0.2000	0.5490	0.9712	41.04	0.8556	0.6083	0.5938
	5	0.8878	0.8857	0.8880	0.1120	0.1143	0.5167	0.9829	61.47	0.8869	0.6217	0.5912
2	1	0.8401	0.3429	0.9073	0.0927	0.6571	0.3333	0.9109	5.11	0.6251	0.2472	0.2471
	2	0.8980	0.7429	0.9189	0.0811	0.2571	0.5532	0.9636	32.74	0.8309	0.5848	0.5763
	3	0.9082	0.8857	0.9112	0.0888	0.1143	0.5741	0.9833	79.52	0.8985	0.6665	0.6454
	4	0.8980	0.8571	0.9035	0.0965	0.1429	0.5455	0.9791	56.16	0.8803	0.6316	0.6099
	5	0.9150	0.8857	0.9189	0.0811	0.1143	0.5962	0.9835	87.83	0.9023	0.6829	0.6650
3	1	0.8776	0.6571	0.9073	0.0927	0.3429	0.4894	0.9514	18.77	0.7822	0.4988	0.4916
	2	0.9218	0.8000	0.9382	0.0618	0.2000	0.6364	0.9720	60.75	0.8691	0.6702	0.6644
	3	0.9150	0.8000	0.9305	0.0695	0.2000	0.6087	0.9718	53.56	0.8653	0.6512	0.6431
	4	0.9116	0.9143	0.9112	0.0888	0.0857	0.5818	0.9874	109.45	0.9127	0.6855	0.6619
	5	0.9218	0.9143	0.9228	0.0772	0.0857	0.6154	0.9876	127.47	0.9185	0.7105	0.6918
4	1	0.9048	0.6857	0.9344	0.0656	0.3143	0.5854	0.9565	31.06	0.8100	0.5797	0.5773
	2	0.9218	0.8286	0.9344	0.0656	0.1714	0.6304	0.9758	68.80	0.8815	0.6801	0.6717
	3	0.9252	0.8571	0.9344	0.0656	0.1429	0.6383	0.9798	85.41	0.8958	0.6994	0.6893
	4	0.9048	0.8857	0.9073	0.0927	0.1143	0.5636	0.9833	75.89	0.8965	0.6586	0.6359
	5	0.8810	0.8857	0.8803	0.1197	0.1143	0.5000	0.9828	57.00	0.8830	0.6081	0.5744
5	1	0.9116	0.7714	0.9305	0.0695	0.2286	0.6000	0.9679	45.19	0.8510	0.6314	0.6247
	2	0.9388	0.8286	0.9537	0.0463	0.1714	0.7073	0.9763	99.49	0.8911	0.7313	0.7283
	3	0.9422	0.8571	0.9537	0.0463	0.1429	0.7143	0.9802	123.50	0.9054	0.7504	0.7463
	4	0.9388	0.8571	0.9498	0.0502	0.1429	0.6977	0.9801	113.54	0.9035	0.7395	0.7344
	5	0.9286	0.9143	0.9305	0.0695	0.0857	0.6400	0.9877	142.81	0.9224	0.7282	0.7127
